# Malignant Tumors Misdiagnosed as Liver Hemangiomas

**DOI:** 10.3389/fsurg.2021.715429

**Published:** 2021-08-11

**Authors:** Murat Baki Yıldırım, İbrahim Tayfun Şahiner, Arzu Poyanlı, Bülent Acunaş, Mine Güllüoǧlu, Cem İbiş, Yaman Tekant, İlgin Özden

**Affiliations:** ^1^Departments of General Surgery, Istanbul Faculty of Medicine, Istanbul University, Istanbul, Turkey; ^2^Departments of Radiology, Istanbul Faculty of Medicine, Istanbul University, Istanbul, Turkey; ^3^Departments of Pathology, Istanbul Faculty of Medicine, Istanbul University, Istanbul, Turkey

**Keywords:** hemangioma, atypical hemangioma, misdiagnosis, liver malignancy, malign mass

## Abstract

**Background and Aim:** To derive lessons from the data of patients who were followed for various periods with the misdiagnosis of liver hemangioma and eventually found to have a malignancy.

**Material and Methods:** The records of 23 patients treated between 2003 and 2018 were analyzed retrospectively.

**Results:** Twelve patients were men and 11 were women; median (range) age was 55 (35–80). The principal diagnostic modality for the initial diagnosis was ultrasonography (*n*:8), magnetic resonance imaging (MRI) (*n*:13), and computed tomography (CT) (*n*:2). At our institution, MRI was performed in 16 patients; the diagnosis was made with the available MRI and CT studies in five and two patients, respectively. In other words, the ultrasonography interpretations were not confirmed on MRI; in others, the MRI or CT examinations were of low quality or they had not been interpreted properly. Fifteen patients underwent surgery; the other patients received chemotherapy (*n*:6) or chemoembolization (*n*:2). The misdiagnosis caused a median (range) 10 (0–96) months delay in treatment. The final diagnoses were hepatocellular carcinoma in 12 patients, cholangiocarcinoma in four patients, metastatic mesenchymal tumor, metastasis of colon cancer, metastatic neuroendocrine carcinoma, sarcomatoid hepatocellular carcinoma, angiosarcoma, thoracic wall tumor, and metastatic tumor of unknown primary in one patient each.

**Conclusions:** High-quality MRI with proper interpretation and judicious follow up are vital for the accurate differential diagnosis of liver lesions.

## Introduction

Hemangioma is the most common liver tumor with an incidence between 1 and 20% (1). The diagnosis is being made with increasing frequency due to the widespread availability of and broadening indications for imaging techniques. Actually, treatment is indicated in a very small subset of patients for pain, enlargement, diagnostic uncertainty, and extremely rare indications such as rupture and Kasabach-Merritt syndrome. The real burden to the health care system is the problem of making accurate diagnoses in a very high number of patients-an issue that entails the risk of missed malignancies. Although the issue has been commonly discussed on an anecdotal basis in hepatopancreatobiliary surgery circles, case reports and only a single series including 28 patients (Portolani) have been published (2–11). Most case reports are actually “clinician's success” stories in which the radiological ambiguity was resolved, usually by biopsy or resection (3–5, 9, 10, 12, 13). Others and the paper by Portolani et al. are on the most probably underreported problem of the patients whose courses took an unfavorable direction due to misdiagnosis (2, 6–8, 11).

In this paper, the institutional experience was analyzed to derive lessons from the data of 23 patients who were followed for various periods with the misdiagnosis of liver hemangioma and eventually found to have a malignancy.

### Aim of the Study

To derive lessons from the data of patients who were followed for various periods with the misdiagnosis of liver hemangioma and eventually found to have a malignancy.

## Materials and Methods

The records of 23 patients who attended the outpatient clinic between 2003 and 2018 were analyzed retrospectively. This is a retrospective study on the data of the patients who were treated at our hospital according to the best practices of the study period. Our IRB waives the need for ethical approval for these observational studies. The research was conducted according to the principles of the World Medical Association Declaration of Helsinki “Ethical Principles for Medical Research Involving Human Subjects” (amended in October 2013).

## Results

Twelve patients were men and 11 were women; median (range) age was 55 (35–80).

Ten of the 23 patients were HBsAg positive and one patient was anti-HCV antibody positive; one other patient had been a moderate-to-heavy drinker for 15 years. The causes of the initial diagnostic errors were summarized in Table 1; the issue was resolved by proper MRI at our institution (*n*:13) or correct interpretation of the available MRI (*n*:5) or CT (*n*:2) studies.

**Table 1:** The causes of the initial misdiagnosis.

Reliance on USG only	8
Inadequate MRI	8
Poor interpretation of the MRI study	5
Poor interpretation of the CT study	2

Fifteen patients underwent surgery. The other eight patients received chemotherapy (*n*:6) or chemoembolization (*n*:2). The misdiagnosis caused a median (range) 10 (0–96) months delay in treatment. The final diagnoses were summarized in Table 2.

**Table 2:** The final diagnoses of the patients.

Hepatocellular carcinoma	12
Cholangiocarcinoma	4
Angiosarcoma	1
Metastasis of colon cancer	1
Metastatic mesenchymal tumor	1
Metastatic neuroendocrine carcinoma	1
Metastatic tumor of unknown primary	1
Sarcomatoid hepatocellular carcinoma	1
Thoracic wall tumor	1

Of the eight patients who were not the candidates for surgery, four had been referred with the wrong diagnoses but there were no significant delays before treatment: hepatocellular carcinoma in an 80-year-old patient with comorbid conditions, hepatocellular carcinoma with peritoneal metastases, metastatic sigmoid colon carcinoma, and metastatic tumor of unknown origin. The histories of the other four patients reveal important lessons for the clinician:

First, the patient who had a neuroendocrine tumor had undergone laparoscopic cholecystectomy 2 years before. The preoperative MRI examination had detected a “hemangioma” in the left lobe; the operation report was not available. Multiple liver masses (largest: 115 mm) and the pancreatic primary tumor were diagnosed at our institution. Second, the patient with giant inoperable cholangiocarcinoma surrounding the superior mesenteric and hepatic arteries and invading the portal vein had been under follow-up for 4 years, with USG only at various centers with the diagnosis of liver hemangioma.

Third, the 44-year-old woman with extensive intrahepatic dissemination of hepatocellular carcinoma had been a known HBV carrier for 2 years but had not been referred to liver imaging until she became symptomatic. She was treated with chemoembolization.

The fourth patient had undergone resection of a 11-cm retroperitoneal tumor; the pathological diagnosis was “mesenchymal tumor with lipomatous areas” and the pathologist stated that there was no evidence of malignancy. In fact, she had multiple but operable metastases that were interpreted as “hemangiomas” on the preoperative USG and “hemangiomas or cystic metastases” on the preoperative CT (the films were not available); the MRI examination recommended by the radiologist had not been performed. The liver lesions were followed by USG for 2 years without apparent growth in size. At 7 years after the operation, she presented with abdominal pain; the metastases had increased in number and the largest liver lesion had grown from 4 to 13 cm. Core biopsy of the liver lesion and examination of the original paraffin sections at the university pathology department revealed mesenchymal tumor with hemangiopericytomatous pattern; the Ki-67 index was 3% in the primary tumor and 20% in the liver metastasis. She was referred to the oncology department.

The two patients with the longest delays in diagnoses (86 and 96 months) were HBV carriers in whom a single mass lesion had been misdiagnosed as a hemangioma (most probably regenerative of dysplastic nodules) and was not followed up properly. Single hepatocellular carcinomas (6 and 3.3 cm, respectively) and were eventually diagnosed by MRI; there were no concomitant hemangiomas.

## Discussion

Misdiagnosis of a malignancy as a hemangioma is a widely acknowledged but most probably underreported issue. There are three aspects:

First, the sheer magnitude of the problem. The total population of the OECD countries exceeds 1 billion people. The reported incidence of liver hemangiomas varies between 1 and 20% (1). In 2015, the average number of CT examinations in the OECD countries was 143 per 1,000 population; the corresponding number for MRI was 64.8 (14). Although these figures include examinations for all locations, it is obvious that the differential diagnosis of at least several hundred thousands of hemangiomas is a major undertaking. The very high (98–99%) but-not-100% sensitivity and specificity of MRI for hemangiomas leave a speciously small error margin that actually poses risks to thousands of patients (15). For the sake of comparison, during the study period of the present report on 23 misdiagnosed cases, liver hemangioma (multiple in some cases) was correctly identified in 1,243 patients (data collected for a another study with the following exclusion criteria: previous treatment for any malignancy (except for well-differentiated thyroid carcinoma), use of vasoconstrictor or anticoagulant drugs, history of liver surgery for any indication and cirrhosis).

Second, the misconception that a proper USG examination is adequate for the diagnosis of a hemangioma and further examination is unnecessary (16, 17). Only 80 % of the liver hemangiomas have a typical appearance on USG (18, 19). Also, hemangiomas undergo various changes (e.g., sclerosis) and their appearances change over time (20).

Third is the distinction between efficacy and effectiveness in imaging. The performance of experienced radiologists using state-of-the-art technology at a university hospital cannot represent the overall performance of an imaging technique under less-than-ideal diverse conditions. The so-called atypical hemangioma may actually be a misdiagnosed hepatocellular carcinoma or cholangiocarcinoma. Therefore, the responsibility of these patients should be assumed by specialized teams including dedicated radiologists (21). Sixteen of the 24 patients in this series had undergone a CT or MRI examination. The quality of the imaging was unacceptable in seven patients. Instead of making a confident diagnosis of a hemangiomas the radiologist should have ordered repeated imaging. In the remaining seven patients with acceptable image quality, the interpretation had been inaccurate. Similarly, in the study by Portolani et al., 75% of the incorrect diagnoses were made at low volume centers. A high-volume center was “characterized by the availability of an interdisciplinary group for the management of liver disease, by a tertiary-level or an academic hospital, or by the certification of almost 40 liver resections per year” (16).

The published literature and the cases reported here lead us to point out the following:

1- A proper contrast-enhanced CT/MRI examination with a state-of-the art apparatus is mandatory for proper characterization of liver lesions, particularly in patients with chronic viral hepatitis (22–25). According to recent data, an MRI examination with a hepatocyte-specific agent is preferred (26, 27).
2- Even the most specific examination method may fail in rare situations (27), especially outside academic centers. The suggestion stated by Portolani et al. (16) to repeat the imaging examination 4–6 months later is a practical safeguard.
3- The clinician should avoid the misconception that “conservative follow up” is always “safe.” Although the radiologist makes the imaging diagnosis, the “overall” diagnosis and responsibility of the patient rests on the clinician.
4- Follow up may be conducted at the same institution (e.g., patients with chronic viral hepatitis) or coordinated with the referring institution or family physician (patients with no or modifiable risk factors such as obesity for chronic liver disease).

A theoretically possible confounding factor is the development of imaging technology during the 15-year study period. However, as reported in Table 1, this may have been the case in at most eight cases (one-third of the patients) in whom inadequate MRI images were the causes of the initial error. Still, the misdiagnosis could be identified at our institution after contrast-enhanced dynamic MRI. In the remaining two-thirds of the patients, reliance on USG only or inadequate interpretation of the available CT or MRI images were implicated.

A limitation of this study is that the data does not allow us to calculate the frequency of misdiagnosis. The patients reported here belong to part of the numerator (there may be other misdiagnosed patients at the initial institutions; the denominator (the total number of patients who received a diagnosis of liver hemangioma at the initial institutions during a particular period) is unknown.

## Conclusion

A multidisciplinary team approach at an experienced center is recommended for a reliable baseline assessment of a liver mass, especially in patients with chronic liver conditions.

## Data Availability Statement

The raw data supporting the conclusions of this article will be made available by the authors, without undue reservation.

## Author's Note

A preliminary report was presented at the 13th Turkish National Congress of HPB Surgery, November 1–4, 2017, Antalya, Turkey.

## Author Contributions

MY, MG, and AP: collecting data and writing. İO: design of study, writing, supervision, and last control of study. YT, Cİ, and BA: supervision. İŞ: collecting analysis data. All authors contributed to the article and approved the submitted version.

## Conflict of Interest

The authors declare that the research was conducted in the absence of any commercial or financial relationships that could be construed as a potential conflict of interest.

## Publisher's Note

All claims expressed in this article are solely those of the authors and do not necessarily represent those of their affiliated organizations, or those of the publisher, the editors and the reviewers. Any product that may be evaluated in this article, or claim that may be made by its manufacturer, is not guaranteed or endorsed by the publisher.
